# Comparison of targeted metagenomics and IS-Pro methods for analysing the lung microbiome

**DOI:** 10.1186/s12866-021-02288-x

**Published:** 2021-08-18

**Authors:** T Goolam Mahomed, RPH Peters, GHJ Pretorius, A Goolam Mahomed, V Ueckermann, MM Kock, MM Ehlers

**Affiliations:** 1 Department of Medical Microbiology, University of Pretoria, Pretoria, South Africa; 2 CAPHRI School for Public Health & Primary Care, Department of Medical Microbiology, Maastricht University Medical Centre, Maastricht, The Netherlands; 3 Synexa Life Sciences, Cape Town, South Africa; 4 Louis Pasteur Private Hospital, Pretoria, South Africa; 5 Department of Internal Medicine, University of Pretoria, Pretoria, South Africa; 6 Department of Medical Microbiology, Tshwane Academic Division, National Health Laboratory Service, Tshwane, South Africa

## Abstract

**Background:**

Targeted metagenomics and IS-Pro method are two of the many methods that have been used to study the microbiome. The two methods target different regions of the 16 S rRNA gene. The aim of this study was to compare targeted metagenomics and IS-Pro methods for the ability to discern the microbial composition of the lung microbiome of COPD patients.

**Methods:**

Spontaneously expectorated sputum specimens were collected from COPD patients. Bacterial DNA was extracted and used for targeted metagenomics and IS-Pro method. The analysis was performed using QIIME2 (targeted metagenomics) and IS-Pro software (IS-Pro method). Additionally, a laboratory cost per isolate and time analysis was performed for each method.

**Results:**

Statistically significant differences were observed in alpha diversity when targeted metagenomics and IS-Pro methods’ data were compared using the Shannon diversity measure (*p*-value = 0.0006) but not with the Simpson diversity measure (*p*-value = 0.84). Distinct clusters with no overlap between the two technologies were observed for beta diversity. Targeted metagenomics had a lower relative abundance of phyla, such as the *Proteobacteria*, and higher relative abundance of phyla, such as *Firmicutes* when compared to the IS-Pro method. *Haemophilus*, *Prevotella* and *Streptococcus* were most prevalent genera across both methods. Targeted metagenomics classified 23 % (144/631) of OTUs to a species level, whereas IS-Pro method classified 86 % (55/64) of OTUs to a species level. However, unclassified OTUs accounted for a higher relative abundance when using the IS-Pro method (35 %) compared to targeted metagenomics (5 %). The two methods performed comparably in terms of cost and time; however, the IS-Pro method was more user-friendly.

**Conclusions:**

It is essential to understand the value of different methods for characterisation of the microbiome. Targeted metagenomics and IS-Pro methods showed differences in ability in identifying and characterising OTUs, diversity and microbial composition of the lung microbiome. The IS-Pro method might miss relevant species and could inflate the abundance of *Proteobacteria.* However, the IS-Pro kit identified most of the important lung pathogens, such as *Burkholderia* and *Pseudomonas* and may work in a more diagnostics-orientated setting. Both methods were comparable in terms of cost and time; however, the IS-Pro method was easier to use.

**Supplementary Information:**

The online version contains supplementary material available at 10.1186/s12866-021-02288-x.

## Background

Microorganisms occur as communities and often play an important role in host metabolism [1–3]. This collective of microorganisms within a community (ecosystem) and their genetic material is referred to as a microbiome [4, 5]. Previously, culture-dependent techniques were used to study the microbiome, however, researchers have found that less than 1 % of all bacteria can be cultured and that the microbiome is often more diverse than culture methods suggest [4, 6]. Culture-independent methods, such as denaturing gradient gel electrophoresis, fluorescence *in situ* hybridisation, microarrays, quantitative polymerase chain reaction and terminal length polymorphisms have since been used to study the microbiome [7–11]. However, the most popular approach to study the microbiome is sequencing analysis, either using Sanger or next-generation sequencing (NGS) technologies using a targeted approach [8–10, 12].

The most popular target of these sequencing methods is the 16 S rRNA gene region [13, 14]. The 16 S rRNA gene is useful for identifying bacteria and determining phylogenetics as this gene is present in all prokaryotes i.e. it is universal, is easily isolated and is highly conserved (i.e. the sequences and the length of the genes change little with time) [9, 15, 16]. The 16 S ribosomal subunit consists of both hypervariable and conserved regions, with the sequencing primers that are commonly used targeting the conserved regions between the hypervariable regions [17, 18]. There are nine hypervariable (V1-V9) regions and nine conserved regions (which alternate) [17, 19]. Among the most common primers used for 16 S rRNA gene are the 27 F and 518R primers that cover the V1 to V3 hypervariable regions [20, 21]. While, the V1-V3 region of the 16 S rRNA was shown to have the highest similarity with full-length sequences of the 16 S rRNA gene, the ideal region of choice has not been agreed upon in the field of targeted metagenomics and is dependent on many factors, such as the sequencing platform used [22]. The different sequencing platforms generate different sized read fragments which impacts the choice of primer, ideally the primers used should generate amplicons with similar size to fragments that can be sequenced by the platform; the Illumina Miseq platform generates fragments of 300 bp and therefore utilises primers that result in short amplicons e.g. 27 F and 518R primers that target V1-V3 region wehereas the PacBio platform generates reads of > 100 000 bp and is therefore able to use primers target the entire 16 S rRNA gene e.g. 27 F and 1429R primers [23, 24].

The IS-Pro method, a method that targets the intergenic spacer (IS) region between the 16 S rRNA and 23 S rRNA was developed by Budding and colleagues in 2010 to identify all bacteria present in the sample i.e. a clinical specimen. The intergenic spacer region was chosen due to its variability; this region is more variable than the hypervariable regions of the 16 S rRNA [25, 26]. The IS region has species-specific differences in length and sequence polymorphisms, which are used to identify bacteria and can be termed a profiling method [25, 26]. This method has been used to study the vaginal microbiome, the gut microbiome and has been tested in a clinical setting (clinical microbiology laboratory) for the identification of bacteria from “sterile” body sites/fluids [25–34].

Studies that have investigated the lung microbiome have mostly used targeted metagenomics. To our knowledge, no studies have used the IS-Pro method to study the lung microbiome. The aim of this study was to compare the IS-Pro method to 16 S rRNA sequencing in its ability to discern the microbial composition of the lung microbiome of COPD patients.

## Methods

### Study design and study participants

Patients suffering from COPD that were admitted or were attending a clinic at one of three hospitals (one academic, one district and one private) in the Tshwane Health District were invited to participate in the study. If the inclusion and exclusion criteria were met and written informed consent was obtained, participants were included in the study (Table S1). Ethical approval was granted from The Research Ethics committee, Faculty of Health Sciences, University of Pretoria (REC no: 237/2017).

### Sputum specimen processing and bacterial DNA extraction

Spontaneously expectorated sputum specimens were collected from all participants at a single time point. The specimens were transported on ice without any preservation media and stored at -80 °C (Innova U535 Upright, Eppendorf, Germany) until batch processing could occur. Each sputum specimen was thawed (after all specimens were collected) and treated with an equal volume of 0.1 % dithiothreitol (DTT) (Roche. Switzerland) (to reduce sputum viscosity) and homogenised for 30 s (Vortex-Genie®2; Scientific Industries Inc., USA) [35–38]. An aliquot of the homogenised sputum (250 µL) was transferred to a new 2 mL microcentrifuge tube (Axygen, Corning, Germany) and centrifuged at 4 000 x *g* (Spectrafuge™ 24D, Labnet International Inc., USA) for 30 min before extraction. Bacterial DNA extraction was performed using the Isolate II Genomic DNA Kit (Bioline, UK). The manufacturer’s instructions were followed with the addition of 10 mg/mL lysozyme (Sigma-Aldrich, USA), 3 U/µL lysostaphin (Sigma-Aldrich, USA) and 6.75 µL of 10 U/µL mutanolysin (Sigma-Aldrich, USA) to the hard-to-lyse buffer [20 mM Tris (Sigma-Aldrich, USA) pH 8.0; 1 % Triton X-100 (Amresco, USA); 2 mM EDTA(Sigma-Aldrich, USA)]. The extracted DNA was separated into three aliquots [in two new 2 mL microcentrifuge tubes (Axygen, Corning, Germany)] and stored at -20 °C (Samsung, South Korea) until further usage. Aliquot 1 was used for targeted metagenomics and aliquot 2 was used for the IS-Pro method. The DNA concentration and purity were measured using the Genova Nano spectrophotometer (Jenway, UK).

### Targeted metagenomics

An aliquot of the extracted bacterial DNA was sent to Inqaba Biotechnical Industries (Pretoria, South Africa), a commercial NGS service provider, for sequencing. Briefly, bacterial DNA was amplified using a PCR targeting the V1-V3 region of the 16S rRNA gene using the 27F and 518R primers [39]. The amplicons generated from the PCR assay were gel purified, end-repaired (removal of 3’ overhangs) and the Illumina-specific adapter sequences were ligated to each amplicon using the NEBNext® Ultra™ II DNA library prep kit for Illumina® (New England Biolabs, USA) according to the manufacturer’s instructions. After ligation (and quantification) the samples were indexed using the NEBNext® Multiplex Oligos for Illumina® ^(^Index Primers Set 1) (New England Biolabs, USA), followed by purification with AMPure XP beads (Beckman Coulter, USA). The purified amplicons were sequenced using the MiSeq v3 platform (Illumina, USA) for 600 cycles. Each sample generated 300 bp paired-end reads. The resulting fastq files were analysed for quality control and were analysed using QIIME2 version 2019.1 (1,548,866,877) and the Greengenes database (13.8) [40]. Only the extracted bacterial DNA was sequenced and analysed, unfortunately due to the high cost of sequencing in South Africa, no negative controls or spiked controls (positive controls) were included. The workflow for the QIIME analysis was as follows: (i) Demultiplex paired-end reads were imported using Casava 1.8 paired-end demultiplexed fastq option, (ii) The sequence quality control and feature table was conducted using the Deblur plugin with sequences truncated at 240 bp, (iii) A Naïve Bayes classifier with Greengenes reference sequences (99 % identity) and the 27 F and 518R primers (target V1 to V3 region of 16 S rRNA) was trained using the q2-feature-classifier option and (iv) taxonomic analysis was performed. No additional analysis, such as the removal of chimeric sequences or OTUs that were present in only single samples were conducted.

### The IS-Pro method to determine the microbiome

The IS-Pro kit (InBiome, the Netherlands) was used to amplify the previously extracted bacterial DNA, according to the manufacturer’s instructions and was performed at Synexa Life Sciences, Cape Town, South Africa. The kit components included two master mixes (PROTEO and FIRBAC), two control vials (one for *Proteobacteria* and one for *Firmicutes*/*Bacteroidetes*) and eMix (reference marker). The PROTEO master mix targets only the *Proteobacteria*, whereas the FIRBAC master mix targets the *Actinobacteria, Bacteroidetes, Firmicutes, Fusobacteria* and *Verrucomicrobia* phyla [26]. In a microtiter plate, for each sample (*n* = 24), the positive control (included in the kit), the negative control [nuclease-free water, (Qiagen, Germany)] and the following was added: 12 µL of PROTEO master mix (supplied with kit) in a well and 12 µL of FIRBAC master mix (supplied with kit) to a separate well. To each well, 8 µL of extracted bacterial DNA was added. The PCR amplification (Applied Biosystems GeneAmp PCR 9700, ThermoFisher Scientific, USA) conditions were as follows: 35 cycles of 94 °C for 30 s, 56 °C for 45 s, and 72 °C for 1 min, followed by a final extension step at 72 °C for 10 min. After amplification, 16 µL of the eMix was added to each well (for the number of samples and controls) in a new microtiter plate and 4 µL of each amplicon was added to a well, followed by denaturation at 94 °C for 3 min. The samples were analysed on the Applied Biosystems 3730xL genetic analyser (ThermoFisher Scientific, USA) at the central analytical facility (CAF) at Stellenbosch University, Cape Town, South Africa.

Data was analysed using the IS-Pro software suite (InBiome, The Netherlands), which generates microbial profiles. The colour of the peak generated is obtained from the labelled primers and provides information of which phyla has been amplified, whereas the length of the fragment obtained is used to identify the bacteria to lower taxonomic levels (genus, species or subspecies). Each peak within a profile is considered an operational taxonomic unit (OTU) and its intensity determined the abundance.

### Statistical analysis and data visualisation

The program used for 16 S rRNA analysis, QIIME2, generated the taxonomy table and OTU table in. qza format. The .qza files were converted into the correct format for phyloseq using the R package, QIIME2R version 0.99.21. The IS-Pro data was converted to the required format for phyloseq manually (in Excel). Phyloseq requires two files to process and analyse data: (i) a file containing the taxonomic information (of the microorganisms) and (ii) a file containing the read/OTU counts present in each sample. The IS-Pro output was a single file that contains both taxonomic and OTU counts and therefore needed to be separated; as such the taxonomic data and the OTU counts were moved into two different files, which was used as the taxonomy table and OTU table, respectively. The data was analysed in R using the following packages: (i) phyloseq version 1.30.0 (alpha diversity, beta diversity, statistical tests, PCoA (Principal component analysis), and relative abundance of the taxa), (ii) ggplot2 version 3.3.2 (for the plotting of all graphs) and (iii) DESeq2 version 1.26.0 (to determine if there was a log2fold difference; note that internal normalisation was performed during the analysis.

### Cost per isolate and time analysis

Targeted metagenomics was compared to the IS-Pro method in terms of cost, time to analysis and user-friendliness. The cost calculated included estimates based on the procurement of resources in our own laboratory at the Department of Medical Microbiology of the University of Pretoria, the cost for sample processing, DNA extraction, reagents for PCR assays and PCR clean-up, consumables and the complete cost of sequencing (based on the quote generated by the company that performed sequencing and includes both labour cost and the benchtop cost). Time to analysis was calculated from the date of sequencing results were received to end of the analysis (including statistical analysis). The user-friendliness was determined based on the authors’ experience with QIIME2 and the IS-Pro proprietary software.

## Results

### Patient demographics

A total of 24 participants were enrolled in the study; 18 males and six females in the age group 50 years to 82 years old (median age was 60 years old). Only one of the participants was HIV-infected. Participants were distributed across the three hospitals as follows: (i) Hospital A (Tertiary Academic Hospital): 16 participants, (ii) Hospital B (District Hospital): one participant and Hospital C (Private Hospital): seven participants. Eighteen of the participants were in the stable state of disease at the time of sampling and six of the participants were in the exacerbated state of disease at the time of sampling.

### Comparison of the data outputs (including operational taxonomic units) between the targeted metagenomics and IS-Pro methods for

The two methods generated different outputs, targeted metagenomics generated a fastq file (from next-generation sequencing) and IS-Pro method generated a fsa file (from fragment analysis). These files were analysed using QIIME2 and the IS-Pro propriety software, respectively; QIIME2 generated a .qza file that is a zipped file that contains a BIOM file along with a provenance file whereas IS-Pro generated an excel file (see section "Statistical analysis and data visualisation" for the explanation for further details). Analysis of the IS-Pro data showed that one of the 24 samples did not meet the quality control requirements with the IS-Pro method as the concentration of the internal size marker was too low. Even though the sample generated data using targeted metagenomics, it was excluded from subsequent analysis.

When the two methods were compared (after converting to phyloseq objects), targeted metagenomics identified 631 OTUs. These OTUS could be divided into 14 phyla, 27 classes, 37 orders, 70 families and 76 genera. The IS-Pro method identified 64 OTUs. These OTUS could be divided into six phyla, 11 classes, 18 orders, 27 families and 35 genera. Table S3 shows the number of OTUs and the number of amplicons generated per sample. The IS-Pro method showed less variation in the number of amplicons between samples, with an IQR of 1. Alternatively, the targeted metagenomics method had IQR of 11129.5 for number of amplicons. The IS-Pro method additionally had less variation in the number of OTUs generated from each sample with IQR of 4 whereas the targeted metagenomics method had an IQR of 70.5.

### Alpha and beta diversity analysis

**Fig. 1:**
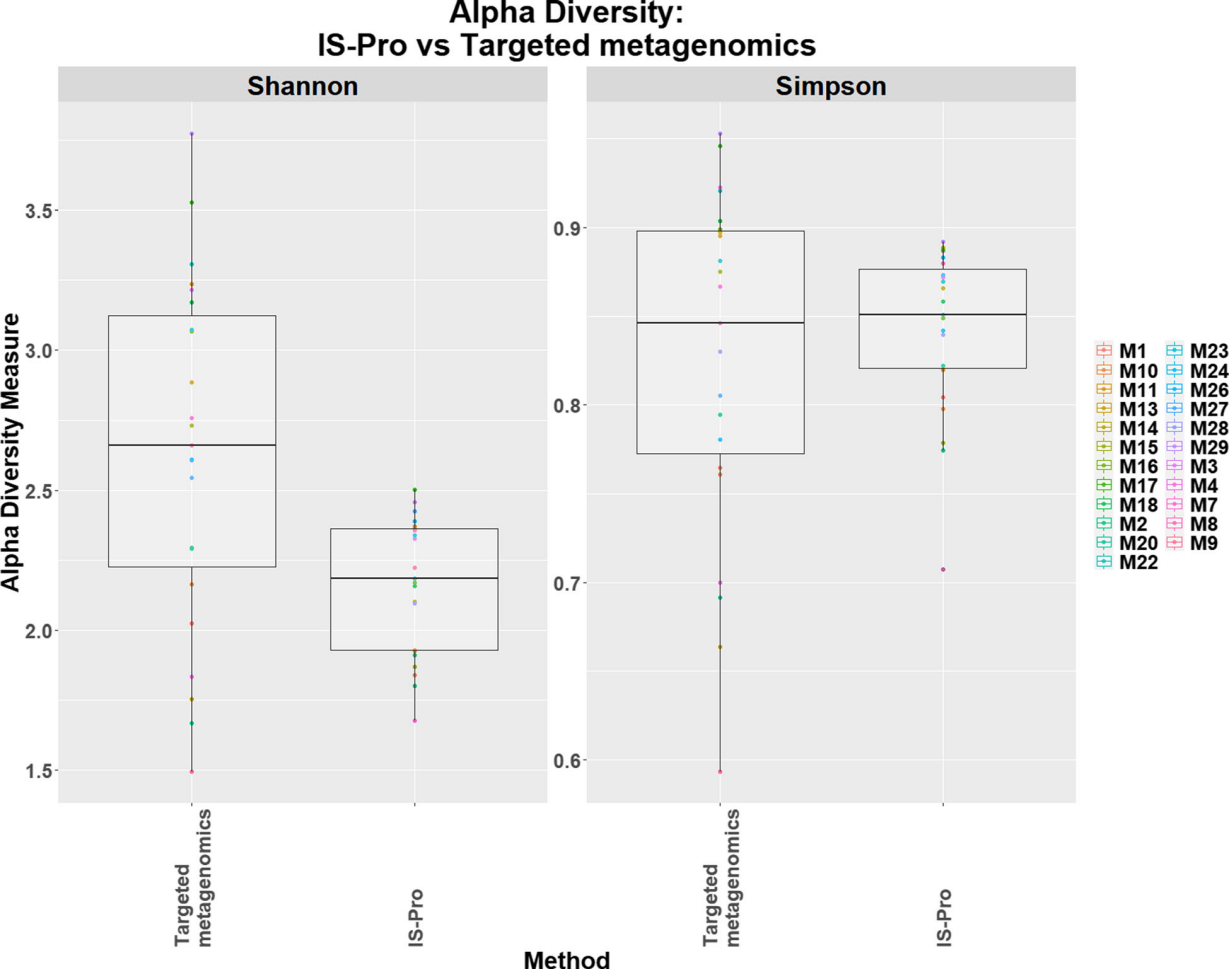
The alpha diversity box-plot of the sputum microbiome of COPD participants comparing targeted metagenomics and IS-Pro methods (*n* = 23) for Shannon and Simpson diversity measures. Each dot on the graph represents a sample. The boxes represent the interquartile range (IQR) and the horizontal line represents the median. The median values for the Shannon diversity measure were as follows: (i) 16 S rRNA sequencing = 2.732 and (ii) IS-Pro method = 2.183. The median values for the Simpson diversity measures were as follows: (i) targeted metagenomics = 0.866 and (ii) IS-Pro method = 0.851

When alpha diversity was compared between targeted metagenomics and IS-Pro methods (Fig. 1), a significant difference was observed using the Shannon diversity measure (using Wilcoxon sum rank test, *p*-value = 0.0006, median values of 2.732 and 2.183); targeted metagenomics showed a higher alpha diversity than the IS-Pro method. No difference was observed with the Simpson diversity measure when comparing targeted metagenomics and IS-Pro methods (using Wilcoxon sum rank test, *p*-value = 0.84, median values of 0.866 and 0.851).

**Fig. 2:**
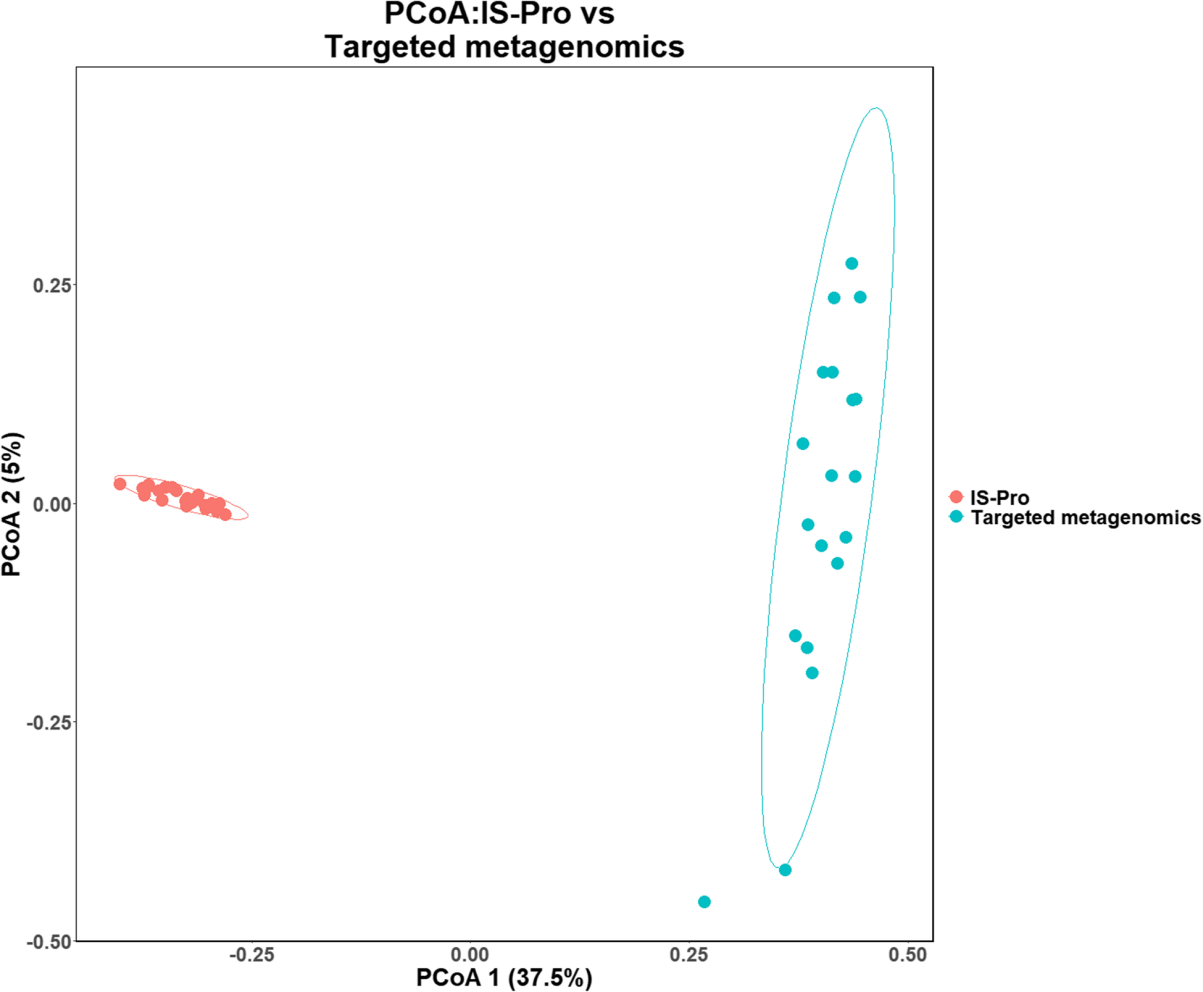
Principal component analysis (PCoA) plot derived using Jaccard diversity measure of the sputum microbiome of COPD participants. The PCoA plot compares targeted metagenomics and IS-Pro methods; with the dots representing each sample

Beta diversity analysis (PCoA analysis) of the two methods (between targeted metagenomics and IS-Pro methods) showed the isolates clustering according to the method (Fig. 2). Both Jaccard diversity and Morisita Horn (not shown) measures showed the two methods forming distinct clusters with no overlap between the two methods. The targeted metagenomics isolates clustered further apart than the IS-Pro method isolates.

### Difference in relative abundance between targeted metagenomics and IS-Pro methods

The most prevalent phyla according to both methods (Table 1 and Figure S1) were *Firmicutes*, *Proteobacteria*, *Bacteroidetes*, *Actinobacteria* and *Fusobacteria*. The IS-Pro method, however, showed a higher relative abundance of the *Proteobacteria, Bacteroidetes* and *Fusobacteria* and lower relative abundance of *Actinobacteria*, and *Firmicutes.*

**Table 1:** Comparison of the relative abundance at a phylum level and genus level for the targeted metagenomics and IS-Pro methods

Taxon	Targeted metagenomics	IS-Pro method	Increase/ Decrease (↑/↓)	
*Phylum level*				
* Firmicutes*	57.1 %	40.5 %		**↓**
* Proteobacteria*	16 %	38 %		**↑**
* Bacteroidetes*	10.3 %	12.4 %		**↑**
* Actinobacteria*	12.3 %	2.5 %		**↓**
* Fusobacteria*	2.3 %	6.6 %		**↑**
*Genus level*				
* Actinomyces*	5.72 %	0.74 %		**↓**
* Burkholderia*	0.00 %	0.82 %		**↑**
* Corynebacterium*	0.11 %	0.78 %		**↑**
* Eikenella*	0.01 %	0.82 %		**↑**
* Escherichia*	0.00 %	0.22 %		**↑**
* Fusobacterium*	0.30 %	6.49 %		**↑**
* Granulicatella*	3.60 %	0.00 %		**↓**
* Lactobacillus*	0.10 %	2.64 %		**↑**
* Leptotrichia*	2.44 %	0.00 %		**↓**
* Micrococcus*	0.00 %	0.30 %		**↑**
* Neisseria*	0.02 %	0.692 %		**↑**
* Oleomonas*	0.003 %	0.00 %		**↓**
* Parvimonas*	0.20 %	0.74 %		**↑**
* Peptostreptococcus*	0.04 %	1.69 %		**↑**
* Proteus*	0.00 %	0.39 %		**↑**
* Pseudomonas*	0.02 %	0.69 %		**↑**
* Serratia*	3.75 %	0.78 %		**↓**
* Veillonella*	4.99 %	0.00 %		**↓**

**Fig. 3:**
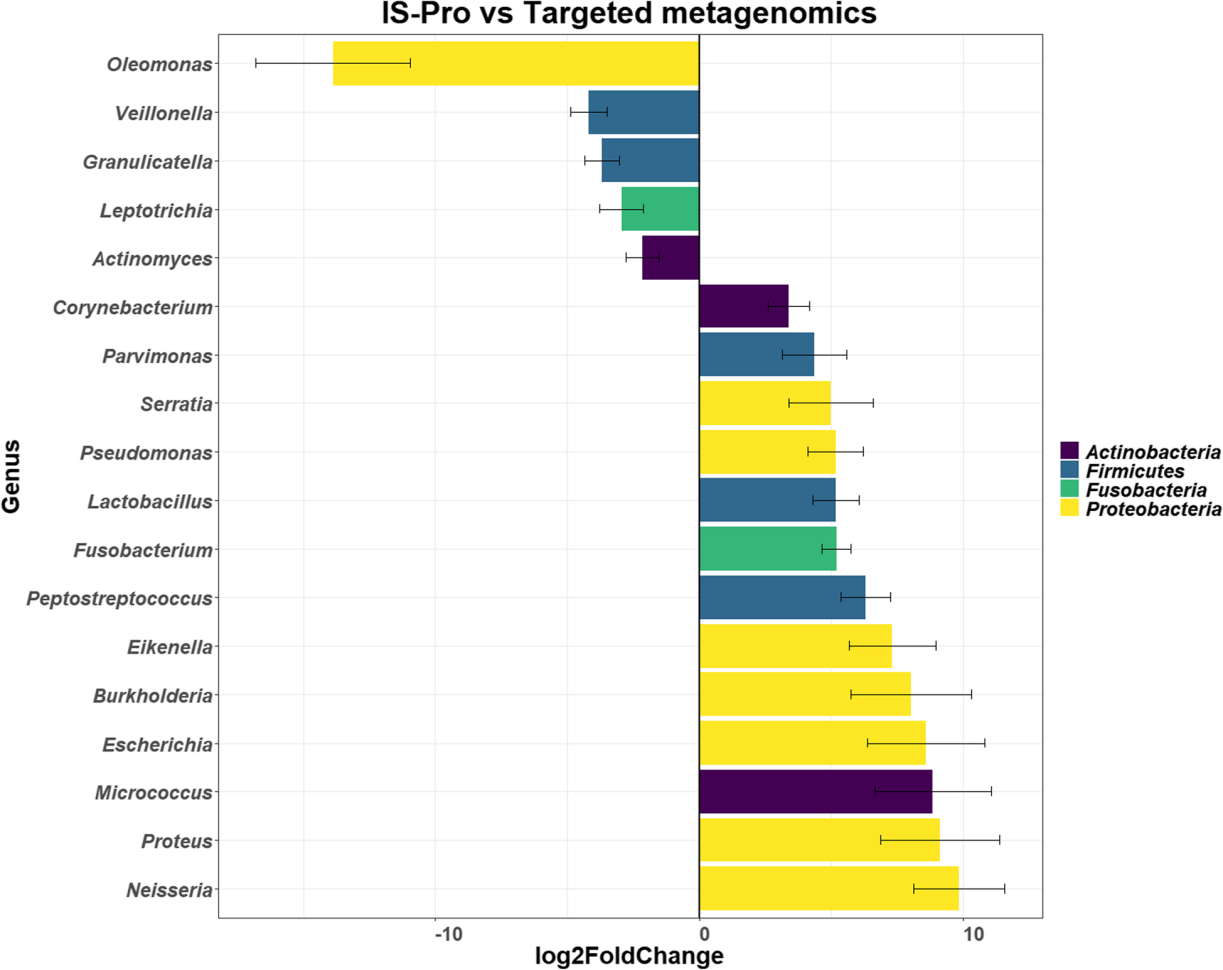
Graph of the DESeq2 analysis showing the log2fold differential abundance of the different genera between targeted metagenomics and IS-Pro methods (*n* = 23) in the sputum microbiome of COPD participants. Differences were considered significant with the *p*-value (adjusted for false discovery rate using Benjamini–Hochberg correction) cut-off of 0.01. Log2fold changes greater than zero indicated an increase in the relevant genera, whereas log2fold changes less than zero indicated a decrease in the relevant genera. All genera above the zero line had an increased relative abundance with the IS-Pro method when compared to targeted metagenomics. The error bars corresponding to the calculated lfcSE (standard error)

A comparison of the relative abundance of targeted metagenomics and IS-Pro methods at genus level showed that the IS-Pro method had an increased abundance of 28 genera including *Burkholderia*, *Fusobacterium*, *Lactobacillus*, *Pseudomonas* and *Peptostreptococcus* and a decreased abundance of 40 genera including *Actinomyces, Veillonella*, *Granulicatella* and *Leptotrichia* (Table 1 and Figure S2). Further analysis showed that the IS-Pro method did not detect any *Veillonella*, *Granulicatella* or *Leptotrichia*. Using DESeq2 (Fig. 3) to compare targeted metagenomics and IS-Pro methods showed a log2fold difference in several genera; with thirteen genera observed in higher abundance with the IS-Pro method and five genera observed in lower abundance with the IS-Pro method. Approximately 50 % (7/13) of the genera that were observed in higher abundances with the IS-Pro method belonged to the *Proteobacteria* phylum and included *Neisseria, Proteus, Escherichia*, *Burkholderia, Eikenella. Serratia* and *Pseudomonas*. Most of the genera that were observed in lower abundances with the IS-Pro method belonged to the *Firmicutes* phylum and included *Veillonella* and *Granulicatella*.

The IS-Pro method was able to classify more OTUs [86 % (55/64)] to a species level than targeted metagenomics, which could classify only 23 % (144/631) of the OTUs to a species level. However, the unclassified OTUs accounted for a higher relative abundance of the IS-Pro method (35 %) than targeted metagenomics (5 %) (Figure S2). The distribution of the unclassified phyla (at class level) for the IS-Pro method was as follows: 16 % for *Firmicutes*, 23 % for *Bacteroidetes* and 61 % for *Proteobacteria* (Figure S3). Although not all the OTUs could be resolved at the genus level for targeted metagenomics, all could be classified at class level (Figure S3).

### Comparison of targeted metagenomics and IS-Pro methods in terms of cost-effectiveness, sample preparation and data analysis

The cost per isolate and time required for each technology is shown below (Table 2). The two technologies were compared in terms of cost, time and user-friendliness of data analysis software.

**Table 2:** Comparison of targeted metagenomics and IS-Pro methods in terms of cost, time and ease of use in our setting

Description	Targeted metagenomics	IS-Pro method
**Laboratory cost per isolate**^a^	$87.57(R 1 441.28)	$117.73 (R 1 937.85)
**Turnaround time (from DNA extraction till statistical analysis)**	9 days (user-dependent and platform-dependent)	7 days
**Hands-on time (labour cost)**	Laboratory: 5 days (1 day for DNA extraction, 4 days for next-generation sequencing and clean-up) Analysis: 4 days (3 days for analysis using QIIME and 1 day for statistical analysis)	Laboratory: 5 days (1 day for DNA extraction, 1 day for the IS-Pro PCR and 1 day for clean-up and 2 days for sequencing) Analysis: 2 days (1 day for analysis using IS-Pro proprietary software and 1 day for statistical analysis)
**Steps involved**	Bacterial DNA extraction PCR amplification of the target region Library preparation (and pooling of samples) Sequencing run Quality control analysis and generation of an OTU table using a program, such as QIIME2. Statistical analysis using a program, such as R	Bacterial DNA extraction PCR amplification using the IS-Pro kit Fragment analysis using a genetic analyser (uses capillary electrophoresis) Analysis of data and generation of an OTU table using IS-Pro proprietary software. Statistical analysis using a program, such as R
**Ease of use**	Requires familiarity with Linux system	Easy to use (requires no prior knowledge of the IS-Pro propriety software)

^a^The cost is the cost at the time the study was conducted, is depicted in South African Rand and is dependent on international exchange rates (the cost in the dollar was based on the exchange rate on 04/10/2020)

Targeted metagenomics and IS-Pro methods are similar in several aspects: (i) both require bacterial DNA extraction and PCR amplification before sequencing and (ii) the cost is similar. However, analysis for targeted metagenomics is more complicated. The targeted metagenomics analysis requires QC analysis followed by clustering of sequences into OTUs and assigning taxonomy to the OTUs. This analysis requires the use of software, such as QIIME2 that is Linux-dependent and requires training to use correctly. The IS-Pro method uses proprietary software that only requires the upload of the sequencing data and the program performs the analysis, thereby requiring no prior knowledge or training.

## Discussion

This study compared targeted metagenomics and IS-Pro methods for its ability to determine the microbial composition of the lung microbiome in COPD patients. A single bacterial DNA extraction was performed for targeted metagenomics and IS-Pro methods to reduce bias. A comparison of targeted metagenomics and IS-Pro methods showed an increased relative abundance of *Proteobacteria* for the IS-Pro method and a difference in alpha diversity and beta diversity between the two methods. This increased abundance was attributed to bacteria, such as *Burkholderia*. Additionally, there was a log2fold difference between targeted metagenomics and IS-Pro methods in the abundance of several *Firmicutes* including *Veillonella*, which may indicate that the IS-Pro method is not optimised to detect *Firmicutes.*

A comparison of the alpha diversity analysis between the two technologies showed a statistically significant difference with the Shannon diversity measure, however, no statistically significant differences were detected using the Simpson diversity measure. The Shannon diversity measure is more sensitive to the number of species i.e. OTUs (richness) than the Simpson diversity measure and favours the less representative taxa as part of the Shannon diversity calculation includes a log-transformation [41]. The IS-Pro method had fewer OTUs than targeted metagenomics in this study and as such, this difference in alpha diversity between the Shannon and Simpson diversity measures is not unexpected. In this study, the beta diversity analysis using PCoA plots showed two distinct clusters (of the same samples) that were associated with the two different technologies. With beta diversity analysis and particularly, cluster-based methods, such as PCoA, the more similar isolates the closer the isolates will cluster [42]. The results of this study can thus be interpreted as follows: (i) The bacterial community structures in targeted metagenomics and IS-Pro methods are distinct i.e. using the same sample, the two methods showed differences between the microbiomes and (ii) with the IS-Pro method, the community structure of samples were more similar to each other (in contrast, targeted metagenomics method showed samples that were more divergent from each other), i.e. targeted metagenomics showed a more diverse microbiome than the IS-Pro method. The alpha diversity and beta diversity results could not be compared to the literature at the time of publication, since there were limited microbiome studies that had performed a direct comparison between targeted metagenomics and IS-Pro methods [27, 43] and none of these studies have reported diversity metrics; to determine if there is a difference in the alpha diversity and beta diversity, direct comparisons are needed.

When the relative abundances profiles of the two technologies were compared, the IS-Pro method showed an increased abundance of the phylum *Proteobacteria* (16.1 % for targeted metagenomics and 38 % for the IS-Pro method). There was only one other published study (by de Meij et al. [27]) that compared targeted metagenomics and the IS-Pro method; however, this study did not observe an increase in *Proteobacteria*. However, this study was conducted using faecal samples of healthy children (*n* = 61) and a different sequencing platform (454 sequencing). The phylum *Proteobacteria* is more commonly associated with disease and inflammation [28, 44]. The increased abundance of the *Proteobacteria* according to the IS-Pro method in this study could be attributed to the use of a master mix that contains primers that select specifically for members of the *Proteobacteria* phylum (PROTEO master mix; part of the IS-Pro kit), which may provide a selective advantage to this phylum [25]. This selective advantage of the master mix was observed for *Fusobacteria* as well (3 % increase using the IS-Pro method).

With both methods, the most abundant phyla were *Firmicutes, Proteobacteria, Bacteroidetes, Actinobacteria* and *Fusobacteria.* Studies on the lung microbiome (both in healthy individuals and those suffering from lung diseases) have observed that these phyla typically dominate the lung microbiome [45, 46]. In the studies that have focused on the individuals suffering from lung disease, the *Proteobacteria* phyla is usually more prevalent [45–50]. However, in this study with both the targeted metagenomics method and the IS-Pro method, the *Firmicutes* phyla was more prevalent. This higher prevalence of *Firmicutes* is usually seen in heathy individuals or those suffering from mild lung disease e.g. mild COPD [49, 50].

At a genus level, the IS-Pro method showed a lower relative abundance for several genera, including *Streptococcus* (15 % decrease), *Actinomyces* (5 % decrease) and *Veillonella* (5 % decrease) and an increased relative abundance of *Fusobacterium* (6 % increase) and *Lactobacillus* (2.5 % increase). Most of the genera that showed increased relative abundance belonged to the *Proteobacteria* phylum, whereas the genera that showed decreased relative abundance belonged mostly to the *Firmicutes* and *Actinobacteria* phyla. Members of the *Proteobacteria* phylum, which had log2fold increased abundance included *Burkholderia*, *Pseudomonas* and *Serratia*. These bacteria are known lung pathogens, although *Burkholderia* is more commonly found in cystic fibrosis (CF) patients than COPD patients [51–56]. However, these pathogens, particularly *Burkholderia* and *Pseudomonas* can also be found as laboratory contaminants [57]. The genera that showed a decreased relative abundance, three phyla were not detected by the IS-Pro method including *Granulicatella* (*Firmicutes*), *Leptotrichia* (*Fusobacteria*) and *Veillonella* (*Firmicutes*). Analysis of the current literature on targeted metagenomics and IS-Pro methods showed that for the same disease (such as irritable bowel disease), targeted metagenomics consistently detected *Veillonella* while the IS-Pro method only detected *Veillonella* in low numbers (or not at all) [26–28, 31, 33, 34, 58]. This limited detection of *Veillonella* with the IS-Pro method in these studies was surprising as most of the studies were conducted on faecal samples (i.e. the gastrointestinal tract) and this genus is a known coloniser of the gastrointestinal tract (as well as the lungs and oral cavity) and has been known to act as an opportunistic pathogen [59, 60]. Based on this analysis, it appears that the IS-Pro method has difficulty in detecting *Veillonella*, which may be due to primer design, the DNA target region or analysis pipeline. A study by Mukherjee et al. [61] provided a possible explanation for this by suggesting that *Veillonella* have multiple different intergenic spacer regions (these bacteria have different ribosomal operons that have different intergenic spacer regions), which may not be easily identifiable by the IS-Pro method analysis software and could be missed [60].

The IS-Pro method was able to identify more OTUs to a species level than targeted metagenomics, however, it showed a higher relative abundance (35 %) of unclassified genera (i.e. OTUs that could not be assigned to a genus) than targeted metagenomics (5 %). Most of the unclassified genera generated by targeted metagenomics could be classified to either a family or order level, however, the unclassified OTUs generated by the IS-Pro method could only be classified to a phylum level. As the current analysis strategy for the IS-Pro method does not include any quality control (QC) steps, these unclassified OTUs may be low quality (short) sequences, chimeras or PCR artefacts [62]. It has been shown that the choice of the polymerase, the region sequenced, the number of PCR rounds, the platform used and even data analysis can affect the error rates with sequencing, however, these factors may affect the IS-Pro method as well even though the IS-Pro method uses capillary electrophoresis [62, 63]. The more errors introduced, the poorer the quality of the data is which affects the downstream analysis and could influence the microorganisms identified [64].

When comparing the cost, time and ease of use of the two technologies, the IS-Pro method performed better than targeted metagenomics; the IS-Pro method was much easier to use (did not require the user to be familiar with Linux, i.e. requires a higher level of expertise) and had a faster turnaround time (7 days compared to 9 days for targeted metagenomics) (see Table 1). Essentially, targeted metagenomics needs a trained microbiologist or bioinformatician to analyse the data, whereas with the IS-Pro method any personnel can perform the analysis. The only disadvantage of the IS-Pro method was the operational cost was slightly more expensive than targeted metagenomics ($117.73 (R 1 937.85) compared to $87.57 (R 1 441.28) per sample).

There are several factors that are known to introduce bias in microbiome studies. These include the primer design, the library preparation, DNA extraction method, the PCR amplification, the sequencing platform and the bioinformatics pipeline [65]. By using the same bacterial DNA (from the same extraction), the authors reduced one bias introducing factor. However, as the two methods are fundamentally different the other types of bias could not be reduced. The primer design was the main bias in this study as different primers that target different regions were used; targeted metagenomics targeted the V1-V3 region of the 16 S rRNA gene using 27 F/518R primers and the IS-Pro method targeted the IS regions between the 16 and 23 S rRNA gene regions using phyla-specific primers (which have additionally introduced bias towards specific phyla). Previous studies have shown when different regions of the 16 S rRNA are targeted there is differences with alpha diversity, beta diversity and taxonomic composition [65, 66]. This suggest that not only targeting different gene regions introduces bias but the gene regions themselves have a bias i.e. by targeted the V1-V3 region an additional bias was introduced. Another potential source of bias is the differing number of cycles at the PCR amplification stage for the two methods; the IS-Pro method amplification stage had 35 cycles whereas the targeted metagenomics amplification stage had 25 cycles. An increased number of PCR cycles has been shown to affect the number of chimeric sequences and increase both PCR artefacts and low-quality sequences without an increased bias towards particular phyla [67]. The two methods used different technologies to generate data and had different outputs (.fsa vs. fastq file) which were analysed using different bioinformatics pipelines (the IS-Pro method used the proprietary software whereas the targeted metagenomics method used QIIME2 with Greengenes database). Both these methods generated taxonomic data that could be compared, however, the steps involved in the bioinformatics analysis could have introduced bias.

One of the major strengths of the IS-Pro method was its ability to identify OTUs to a species level. The IS-Pro method identified a higher proportion of OTUs to a species level and this is most likely due to the use of phyla specific primers and the database used with the proprietary software; however, the proportion may be biased due to the lower number of OTUs identified by the IS-Pro method. Conversely, one of the major weaknesses of the IS-Pro method is that it had a higher number of unclassified sequences. These unclassified sequences could be due a lack of QC steps in the IS-Pro analysis or increased error rates that result in chimera sequences. One of the major strengths of targeted metagenomics was the number of phyla that it identified. The targeted metagenomics method was able to identify 14 different phyla compared to the six phyla identified by the IS-Pro method; this increased number of phyla is most likely due to the primer design as the IS-Pro method primers were designed for specific phyla only (i.e. the IS-Pro method is bias towards these phyla). The major weakness of the targeted metagenomics method is that analysis requires trained personnel who has knowledge of both the experiment and the expected output. Additionally, analysis with targeted metagenomics method can be done using different pipelines or with different databases, each which can affect the output of the analysis. Alternatively, by using the proprietary software with the IS-Pro method this variation is eliminated (which might make it easier to use in a diagnostic setting). Furthermore, the IS-Pro method had less variation between samples for both the number of amplicons and the number of OTUs with IQRs of 1 and 4, respectively; conversely the targeted metagenomics method had IQRs of 11129.5 and 70.5, respectively.

Although this study had a small sample size and only studied a single disease, it provided a detailed comparison of targeted metagenomics and IS-Pro methods. Additionally, this was the first study to perform a direct comparison between targeted metagenomics and IS-Pro methods on sputum specimens. However, an important limitation of this study was that no negative controls (to determine if there was laboratory contamination) or positive controls (spiked samples with known bacteria) were used. The lack of negative controls e.g. DNA extraction negative control means that laboratory contamination with other bacteria cannot be ruled out and tools, such as Decontam R cannot be used to remove any potential contamination. The targeted metagenomics was able to detect more OTUs than the IS-Pro method and as a result, showed a more diverse microbiome population; however, these results could not be compared with other literature as there have been no studies that have performed a direct comparison between targeted metagenomics and IS-Pro methods. The targeted metagenomics and IS-Pro methods showed distinct communities for the same sample. Additionally, the IS-Pro method showed an overabundance of phyla, such as *Proteobacteria* and underabundance of phyla, such as *Actinobacteria* and missed several genera that were identified using targeted metagenomics. These differing abundances were postulated to be the result of the IS-Pro kit design (primers that offered a selective advantage) and analysis software (lack of QC). However, while targeted metagenomics performed better than the IS-Pro method for the identification of the lung microbiome in this study [and gastro-intestinal microbiome in other studies (based on indirect comparisons]) and was less costly, the IS-Pro method was easy to perform and analyse (using the propriety software) without any extensive training, had a shorter turnaround time. Based on the fact the IS-Pro method can miss relevant species, such as *Veillonella* and had more OTUs that could not be classified at a family level, a new IS-Pro kit with additional primers (for the amplification of *Veillonella*) and updated analysis software (with QC steps included), could result in an improved kit. The authors suggest that targeted metagenomics be used for research (as it had less bias towards certain phyla and genera) and the IS-Pro method be used as a diagnostic tool in clinical laboratories as it was able to identify most of the important clinical pathogens, such as *Pseudomonas* (especially those found in the lung) and is easy to perform (the test can be conducted by any technician/technologist). However, due to the current pricing, the authors suggest the kit only be used in complicated cases or reference laboratory). However, as the IS-Pro method has been shown to be bias towards *Proteobacteria* (increased prevalence), the results from the IS-Pro method should be interpreted along with the clinical presentation of the patient, as some *Proteobacteria*, such as *Pseudomonas* have been shown to occur as laboratory contamination. Future studies that compare targeted metagenomics and IS-Pro methods should include: (i) different microbiome e.g. oral microbiome and skin microbiome, (ii) use different primers set for the amplification of the 16 S rRNA gene (to compare to the IS-Pro method) e.g. use primers that target the V4 region and (iii) include a larger study population, preferably including different diseases.

## Conclusions

The targeted metagenomics and the IS-Pro methods showed differences in their abilities to identify and characterise OTUs as well as in the diversity and microbial composition of the lung microbiome. The IS-Pro method might miss relevant species and could over-inflate the abundance of members of the *Proteobacteria.*, However, the IS-Pro kit was able to identify most of the important lung pathogens, such as *Burkholderia* and *Pseudomonas* and may work well in a more diagnostics-orientated setting. Both methods were comparable in terms of cost and time; however, the IS-Pro method was easier to use.

## Supplementary Information

**Additional file 1: Table S1. **Inclusion and exclusion criteria for COPD patients in this study. **Table S2.** Clinical characteristic of patients. **Table S3.** Comparison of the number of amplicons and operational taxonomic units for each sample for the targeted metagenomics and IS-Pro methods. **Figure S1.** Relative abundance of specific phyla in the sputum microbiome of COPD participants as detected by targeted metagenomics and IS-Pro methods (*n *= 23). The dots represent the different abundances of each sample, according to the different phyla. Phyla that are depicted with a single line on the y-axis were not present in any samples for that method. **Figure S2.** Bar plots showing the relative abundance of genera in the sputum microbiome of COPD participants as characterised by targeted metagenomics and IS-Pro methods (*n* = 23). The operational taxonomic units that could not be classified at a genus level are indicated as NA on the graph. **Figure S3.** The distribution of the unclassified operational taxonomic units (OTUs) at a class level of the sputum microbiome of COPD participants for targeted metagenomics and IS-Pro methods by phyla. At a class level, all the OTUs from targeted metagenomics could be classified.

## Data Availability

The sequencing data from this study is available in the NCBI Sequence Read Archive (SRA) database (https://www.ncbi.nlm.nih.gov/sra) Bioproject PRJNA683885 (Accession numbers SAMN17041381 to SAMN17041404).

## Abbreviations

16S rRNA: 16 S ribosomal ribonucleic acid
ART: Anti-retroviral therapy
cDNA: Complementary deoxyribonucleic acid
CDQ: Chronic obstructive pulmonary disease questionnaire
CF: Cystic fibrosis
COPD: Chronic obstructive pulmonary disease
DNA: Deoxyribonucleic acid
DNase: Deoxyribonuclease
dsDNA: Double stranded deoxyribonucleic acid
dsRNA: Double stranded ribonucleic acid
DTT: Dithiothreitol
FEV1%: Percentage of the forced vital capacity
HIV: Human immunodeficiency virus
IQR: Interquartile range
IS: Intergenic spacer
IS-Pro: Intergenic spacer profiling
N/A: Not available
NGS: Next generation sequencing
NICD: National Institute for Communicable Diseases of South Africa
OTUs: Operational taxonomic units
PCoA: Principal component analysis
QC: Quality control
QIIME2: Quantitative insights into microbial ecology 2
REC: Research ethics committee
RNA: Ribonucleic acid
rRNA: Ribosomal ribonucleic acid
RT-PCR: Reverse transcriptase polymerase chain reaction
ssDNA: Single stranded deoxyribonucleic acid
ssRNA: Single stranded ribonucleic acid
TB: Mycobacterium tuberculosis
UK: United Kingdom
USA: United States of America
