# Supplementary material for: Comparison of targeted metagenomics and IS-Pro methods for analysing the lung microbiome
Source: BMC Microbiol. 2021 Aug 18;21:228. doi: 10.1186/s12866-021-02288-x (PMC8371770; doi:10.1186/s12866-021-02288-x)
Supplement: Supplementary file 1 — Additional file 1: Table S1. Inclusion and exclusion criteria for COPD patients in this study. Table S2. Clinical characteristic of patients. Table S3. Comparison of the number of amplicons and operational taxonomic units for each sample for the targeted metagenomics and IS-Pro methods. Figure S1. Relative abundance of specific phyla in the sputum microbiome of COPD participants as detected by targeted metagenomics and IS-Pro methods (n = 23). The dots represent the different abundances of each sample, according to the different phyla. Phyla that are depicted with a single line on the y-axis were not present in any samples for that method. Figure S2. Bar plots showing the relative abundance of genera in the sputum microbiome of COPD participants as characterised by targeted metagenomics and IS-Pro methods (n = 23). The operational taxonomic units that could not be classified at a genus level are indicated as NA on the graph. Figure S3. The distribution of the unclassified operational taxonomic units (OTUs) at a class level of the sputum microbiome of COPD participants for targeted metagenomics and IS-Pro methods by phyla. At a class level, all the OTUs from targeted metagenomics could be classified. [file 12866_2021_2288_MOESM1_ESM.docx]

Supplementary Materials and Results

Table S1: Inclusion and exclusion criteria for COPD patients in this study

| **Stable state** | |
| --- | --- |
| **Inclusion criteria** | **Exclusion criteria** |
| HIV patients on antiviral therapy (ART) | Active tuberculosis infection (receiving treatment) |
| Over 40 years of age | Receiving immunosuppressants |
| Able to provide informed consent | Cancer |
|  | Lung surgery within the last six months |
|  | Unable to answer questionnaire (CDQ) |
|  | Antibiotics within last month |
| **Exacerbated state** | |
| **Inclusion criteria** | **Exclusion criteria** |
| HIV patients on antiviral therapy (ART) | Active tuberculosis infection (receiving treatment) |
| Over 40 years of age | Receiving immunosuppressants |
| Able to provide informed consent | Cancer |
| Increased/worsening of respiratory symptoms 48 h prior to visit | Lung surgery within the last six months |
|  | Unable to answer questionnaire (CDQ) |
|  | Unable to give informed consent |
|  | Antibiotics therapy 24 h prior to admission |
|  | Antibiotic therapy administered for more than 12 h after admission |

FEV1% - The ratio of FEV_1_ (forced expiratory volume in 1 second, the amount of air that can be blown out after a second) to FVC (forced vital capacity, the amount of air that can be blown out after a full inspiration)

Table S2: Clinical characteristic of patients

| **Patient** | **Disease State** | **Year** | **HIV status** | **Hospital** | **Smoking status** | **Weather affect cough** | **phlegm without a cold** | **phlegm in morning** | **wheezing** | **allergies** | **Previous TB diagnosis** | **flu vaccine this year** | **worked in mine** |
| --- | --- | --- | --- | --- | --- | --- | --- | --- | --- | --- | --- | --- | --- |
| **S1** | Stable | 2017 | Negative | 1 | Yes | Yes | Yes | Yes | Often | Yes | No | No | No |
| **S2** | Stable | 2017 | Negative | 1 | Yes |  | Yes | Yes | Often | Yes | No | Yes | No |
| **S3** | Stable | 2017 | Negative | 1 | Yes | Yes | Yes | Yes | Never | No | No | Yes | Yes |
| **S4** | Stable | 2018 | Negative | 1 | Stopped | Yes | Yes | No | Often | Yes | No | No | No |
| **S7** | Exacerbation | 2018 | Negative | 1 | Yes | Yes | Yes | Yes | Often | No | No | Yes | No |
| **S8** | Stable | 2018 | Negative | 1 | Yes | Yes | Yes | Yes | Sometimes | No | Yes | No | Yes |
| **S9** | Stable | 2018 | Negative | 1 | No | Yes | No | Yes | Often | Yes | No | No | Yes |
| **S10** | Stable | 2018 | Negative | 1 | Yes | No | Yes | Yes | Often | No | No | No | No |
| **S11** | Exacerbation | 2018 | Negative | 1 | No | Maybe | No | No | Sometimes | No | No | No | No |
| **S13** | Exacerbation | 2018 | Negative | 1 | Stopped | Maybe | Yes | No | Never | No | No | No | No |
| **S14** | Exacerbation | 2018 | Negative | 2 | Yes | Yes | Yes | Maybe | Often | No | No | No | No |
| **S15** | Stable | 2018 | Negative | 1 | Stopped | Yes | Maybe | Maybe | Sometimes | No | No | No | No |
| **S16** | Stable | 2018 | Negative | 1 | No | Yes | No | No | Often | No | No | Yes | No |
| **S17** | Stable | 2018 | Negative | 1 | Stopped | Yes | Maybe | Maybe | Sometimes | No | No | No | No |
| **S18** | Stable | 2018 | Negative | 1 | Stopped | Yes | Yes | Yes | Often | No | No | No | No |
| **S20** | Stable | 2018 | Positive | 3 | Stopped | No | Yes | No | Sometimes | No | Yes | No | No |
| **S22** | Stable | 2018 | Negative | 1 | Stopped | Yes | Maybe | Yes | Often | No | No | No | No |
| **S23** | Stable | 2018 | Negative | 3 | Yes | No | Yes | Yes | Never | No | No | No | No |
| **S24** | Exacerbation | 2018 | Negative | 3 | Stopped | Yes | Yes | Yes | Often | No | Yes | No | No |
| **S26** | Stable | 2018 | Negative | 3 | Stopped | Yes | Ys | Yes | Often | Yes | No | No | No |
| **S27** | Stable | 2018 | Negative | 3 | No | Yes | Yes | Yes | Never | No | No | No | Yes |
| **S28** | Stable | 2018 | Negative | 3 | Yes | Yes | Yes | Yes | Often | No | No | No | No |
| **S29** | Stable | 2019 | Negative | 3 | Stopped | No | Yes | Yes | Sometimes | No | No | Yes | No |

Table S3: Comparison of the number of amplicons and operational taxonomic units for each sample for the targeted metagenomics and IS-Pro methods

| **Sample** | **No of Amplicons:**  **IS-Pro** | **No of OTUs:**  **IS-Pro** | **No of Amplicons:**  **Targeted metagenomics** | **No of OTUs:**  **Targeted metagenomics** |
| --- | --- | --- | --- | --- |
| **S1** | 100 | 10 | 8393 | 54 |
| **S2** | 101 | 12 | 16444 | 49 |
| **S3** | 101 | 16 | 17495 | 145 |
| **S4** | 100 | 15 | 38629 | 111 |
| **S7** | 102 | 13 | 8942 | 110 |
| **S8** | 100 | 16 | 18765 | 130 |
| **S9** | 101 | 17 | 17792 | 110 |
| **S10** | 101 | 10 | 14750 | 67 |
| **S11** | 99 | 12 | 27401 | 211 |
| **S13** | 99 | 16 | 29292 | 116 |
| **S14** | 101 | 11 | 25916 | 78 |
| **S15** | 101 | 12 | 21349 | 142 |
| **S16** | 100 | 15 | 21119 | 179 |
| **S17** | 102 | 19 | 22457 | 176 |
| **S18** | 100 | 13 | 21278 | 218 |
| **S20** | 99 | 10 | 33363 | 110 |
| **S22** | 99 | 16 | 21627 | 175 |
| **S23** | 102 | 15 | 24893 | 179 |
| **S24** | 100 | 15 | 13987 | 96 |
| **S26** | 102 | 17 | 10701 | 145 |
| **S27** | 102 | 19 | 11104 | 149 |
| **S28** | 101 | 13 | 2187 | 63 |
| **S29** | 98 | 18 | 2059 | 87 |
| **Mean** | 100,48 | 14,35 | 18693,17 | 126,09 |
| **Median** | 101 | 15 | 18765 | 116 |
| **Min** | 98 | 10 | 2059 | 49 |
| **Q1** | 100 | 12 | 12545,5 | 91,5 |
| **Q2** | 101 | 15 | 18765 | 116 |
| **Q3** | 101 | 16 | 23675 | 162 |
| **Max** | 102 | 19 | 38629 | 218 |
| **IQR** | 1 | 4 | 11129,5 | 70,5 |


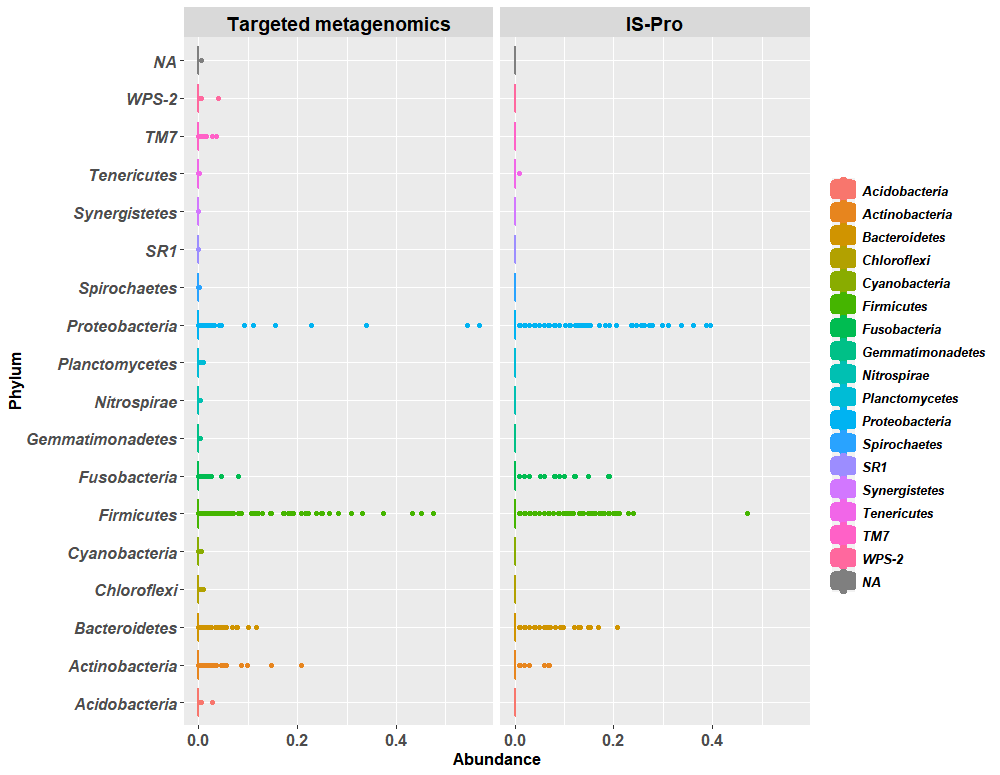


Figure S1: Relative abundance of specific phyla in the sputum microbiome of COPD participants as detected by targeted metagenomics and IS-Pro methods (n=23). The dots represent the different abundances of each sample, according to the different phyla. Phyla that are depicted with a single line on the y-axis were not present in any samples for that method.


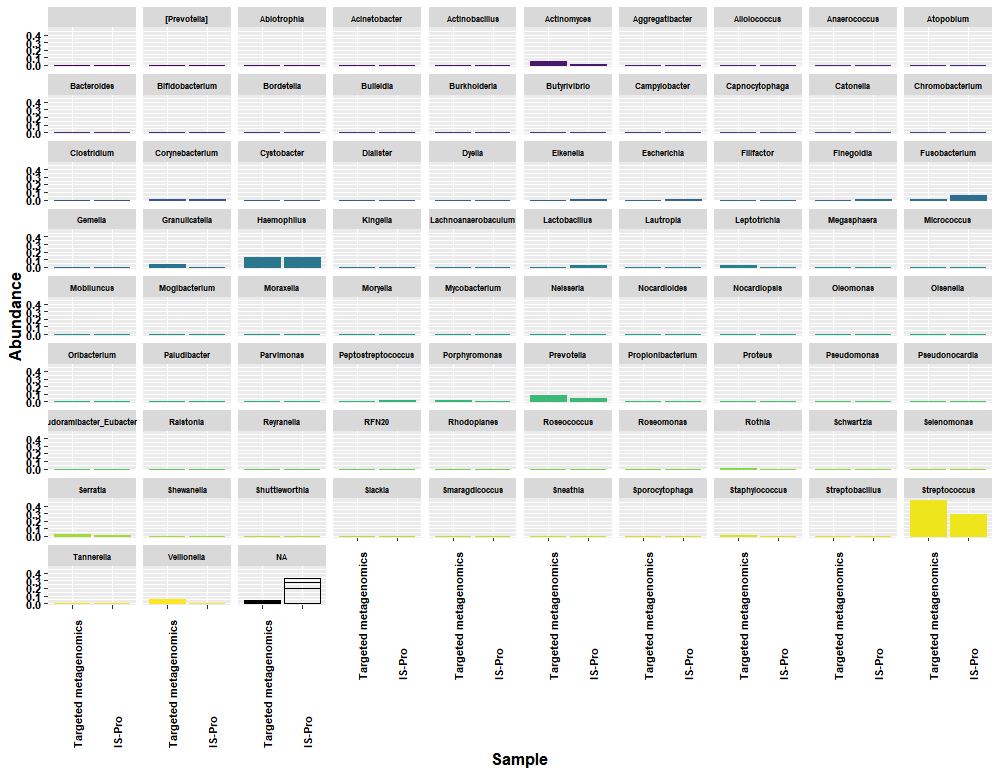


Figure S2: Bar plots showing the relative abundance of genera in the sputum microbiome of COPD participants as characterised by targeted metagenomics and IS-Pro methods (n=23). The operational taxonomic units that could not be classified at a genus level are indicated as NA on the graph.


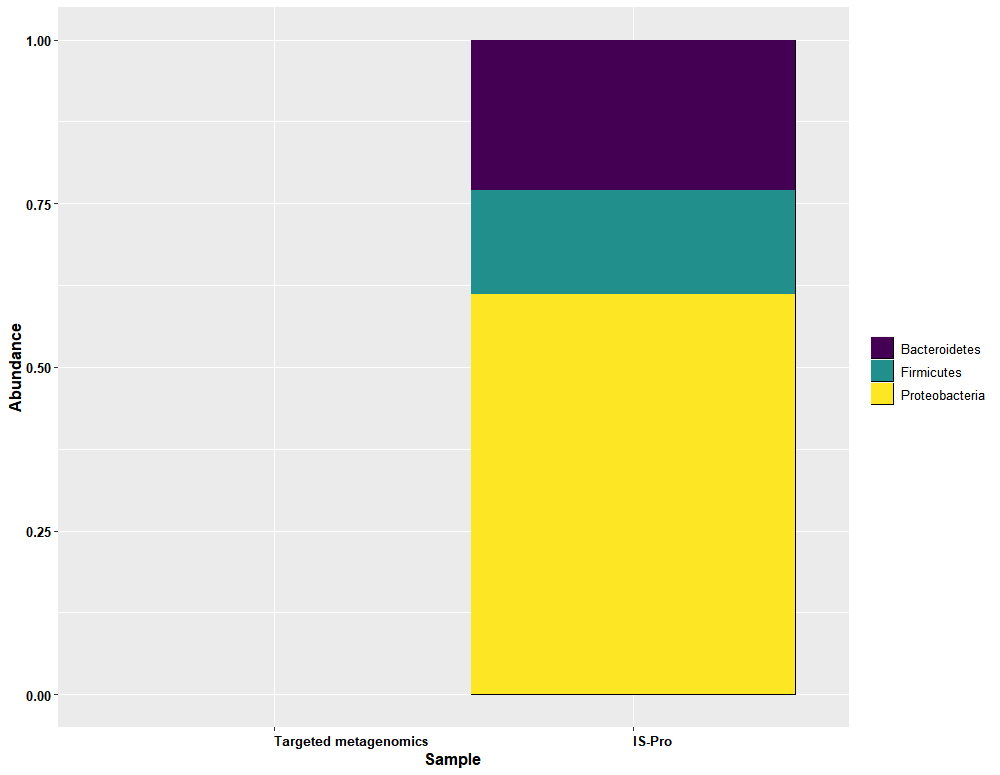


Figure S3: The distribution of the unclassified operational taxonomic units (OTUs) at a class level of the sputum microbiome of COPD participants for targeted metagenomics and IS-Pro methods by phyla. At a class level, all the OTUs from targeted metagenomics could be classified.
